# Entropy-Based Fuzzy TOPSIS Method for Investment Decision Optimization of Large-Scale Projects

**DOI:** 10.1155/2022/4381293

**Published:** 2022-07-18

**Authors:** Junli Cao, Fangfang Xu

**Affiliations:** School of Management, Xi'an University of Architecture and Technology, Xi'an, Shaanxi 710055, China

## Abstract

Investment of large-scale projects must consider various factors, such as economic conditions and investment environment when making decisions. In large-scale project investment problems, almost 90% of them are completed in a multiobjective context. To this end, the authors of this paper have proposed an entropy-based fuzzy TOPSIS synthesis method to assist in the decision-making optimization of large-scale project investment. Firstly, in accordance with the background of the development of multiattribute decision-making method (MADM), closely related to large-scale project investment, the related research works were studied, and the relevant methods are sorted out. Then, the improved vague-entropy-weight based fuzzy TOPSIS (VEWF-TOPSIS) method, entropy and interval language intuitionistic fuzzy sets based TOPSIS (EILIF-TOPSIS) method, and information entropy attribute importance based intuitionistic fuzzy TOPSIS (IEAI-IF-TOPSIS) method were introduced, and a synthesis MADM method that comprehensively combines the above three methods was proposed. Finally, a numerical case was constructed to properly show the effectiveness of the method proposed in the present work, and the conclusions were put forward for providing ideas and methods for large-scale project investment decision-making. It is of great significance that the proposed framework would make large-scale project investment decisions more reasonable and practical, which enriches the research methods of MADM problems to a certain extent and can provide reference for the actual large-scale project investment decision-making problems.

## 1. Introduction

**Figure 1:**
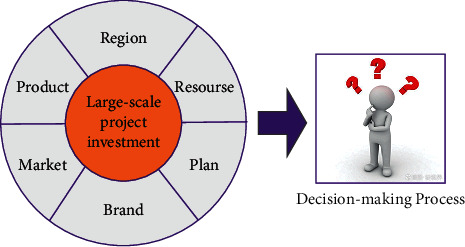
MADM process for large-scale project investment.

Large-scale projects refer to the basic construction projects or social public welfare construction projects that the government makes direct or indirect investment to serve the public and produce social public interests [1, 2]. Different from ordinary projects, investment process for a large-scale project is restricted by social public interests and public will when making decisions. About 90% of large-scale projects are completed under the multiobjective background [3], which means that. in addition to longitudinal analysis of the factors affecting the construction of a single project, the government departments need to compare the benefit and risk indicators of each project to determine the investment priority of multiple projects, as shown in Figure 1. This requires the government to make the best decision scientifically and reasonably when facing the investment of multiple large-scale projects. Due to the lack of scientific and reliable decision-making indicators as well as the limitations of the existing decision-making mechanism, the government has greater blindness and arbitrariness in the investment decision-making of large-scale projects, making the projects face many risks or failing to achieve the expected benefits after completion, or even degrading, resulting in an “white-elephant project” that wastes manpower and money, thereby intensifying social contradictions and seriously damaging the government's image.

MADM problems widely exist in the realms of management, economy, municipal engineering [4, 5], etc. The essence of the problems lies in using the existing decision-making information to sort a set of alternatives and select the best in a certain way. Usually, in the actual process, decision-making problem itself has the property of ambiguity and uncertainty, and the parameters such as the attribute value and attribute weight coefficient of the scheme are uncertain and incomplete, so the fuzzy MADM problem has become a current research hotspot [6, 7].

For handling the uncertainty and fuzziness of data, Zadeh [8] proposed the concept of fuzzy set. Fuzzy set has membership degree but is difficult to describe the degree of denial of evaluation information by decision makers. Therefore, based on the concept of fuzzy set that Zadeh developed, Atanassov proposed an intuitionistic fuzzy set (IFS); that is, the nonmembership degree is added to the fuzzy set in the concept of IFS [9, 10]. The IFS can simultaneously take into account the degree of affirmation and denial of the decision makers to the evaluation information and, at the same time, implicitly consider the degree of hesitation of the decision makers. IFNs have been recognized in decision-making realm widely, and many experts have carried out related research on intuitionistic fuzzy numbers. However, IFNs can only describe the evaluation information in real numbers form. Generally, the decision-making environment has the property of complexity, and the decision-making process of an investor has the property of ambiguity, so that the information related to the process of decision-making is often vague and uncertain. Sometimes, real numbers that are used to express membership and nonmembership are very difficult. Therefore, Atanassov proposed the concept of interval-valued intuitionistic fuzzy set (iIFS); that is, rather than real numbers, in his research work, the membership and nonmembership degrees are represented by interval numbers [11]. Compared with IFNs, interval-valued intuitionistic fuzzy numbers (iIFNs) can better deal with the uncertainty of decision-making information and ambiguity. However, the evaluation information that can be solved by interval intuition fuzzy numbers can only be quantitative information, but the qualitative evaluation information cannot be processed [12, 13].

Entropy is an important concept in thermodynamics. Zadeh first introduced the concept of entropy into the field of decision-making and used the entropy value to express the uncertainty and fuzziness of information. Burillo and Bustince [14] defined intuition fuzzy entropy to represent the hesitation degree of IFSs. However, these formulas can only describe the uncertainty or fuzziness of decision-making information but cannot describe the lack of interval intuitionistic fuzzy information. To this end, Xie and Lv [15] proposed a new formula for interval intuitionistic fuzzy entropy. The new entropy formula takes into account the uncertainty of iIFSs and the information uncertainty degree. The greater the uncertainty, and the more the information missing (that is, the greater the hesitation degree), the greater the corresponding interval intuition fuzzy entropy.

**Figure 2:**
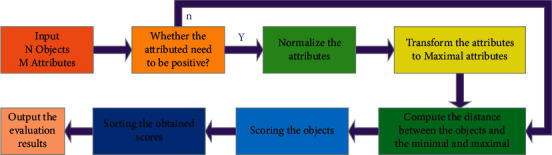
Working flow of the TOPSIS method.

MADM methods include TOPISIS [16–18], ELECTRE [19, 20], gray projection [21], VIKOR [22], and gray correlation degree [23]. In the above-mentioned methods, TOPSIS method is more widely utilized in the realm of MADM problems [24]. The essence of TOPSIS method lies in the fact that, in view of the existence of multiple indicators and multiple schemes, the scheme evaluation and analysis method is to judge the pros and cons of each scheme in the data according to the existing data [25]. Liu et al. [26] have proposed an integrated multiattribute group decision-making (MAGDM) framework, which is developed to assess the new energy investment problems. Keikha [27] utilized GHFNs to model the uncertainty of the assessment values of options against criteria in (MADM) problems. It means that all of the elements of decision matrix are GHFNs. Then, the technique for order of preference is by similarity to ideal solution (TOPSIS) method. The working flow of the TOPSIS method is depicted in Figure 2.

However, in real-world applications, model uncertainty and subjectivity of expert experience exist commonly. In the methods of VEWF-TOPSIS, EILIF-TOPSIS, and IEAI-IF-TOPSIS, there are certain fuzziness and uncertainty in both the model selection and the weight interval given by experts. Therefore, the simple application of any one of the above-mentioned methods will bring unpredictable risks to the investment decision-making of large-scale projects. To this end, the motivation of the present work lies in the urgent need of a comprehensive framework that can take full advantage of the existing TOPSIS-based methods to handle the MADM problems in large-scale project investment in order to maximize the profits. The contributions of the present research are listed as follows:

1. This paper has deeply analyzed the influencing factors of the investment decision-making process of large-scale projects.
2. The existing TOPSIS-based MADM methods have been sorted out, and the problems were summarized.
3. The synthesis method proposed in this paper can comprehensively utilize the results obtained by the above-mentioned three existing methods to output a more objective and reasonable investment decision for a specific large-scale project.
4. Suggestions for real-world application on large-scale project investment have been made.

The remaining content of this research work is organized as flows. In Section 2, the basic property and working flow of three MADM methods, that is, VEWF-TOPSIS, EILIF-TOPSIS, and IEAI-IF-TOPSIS, are detailed, and the proposed synthesis method is given by the end of Section 2. In Section 3, we use a numerical case to show the effectiveness of the proposed method, and the conclusions are drawn in Section 4.

## 2. Entropy-Based Fuzzy TOPSIS Synthesis Method for Decision-Making Problems

### 2.1. VEWF-TOPSIS method

#### 2.1.1. Vague Set and Its Calculation

**Definition 1 (see [24]).**

Let *P*={*p*_1_, *p*_2_, *p*_3_,…, *p*_*n*_} be a space of points (objects), any element of which is represented by *p*, and we use a true membership function *τ*_*A*_(*p*) and a false membership function *λ*_*A*_(*p*) to represent a vague set in space *P.τ*_*A*_(*p*) is the lower bound of the positive membership supporting *p*, *λ*_*A*_(*p*) is the lower bound of the negative membership against *p*, *t*_*A*_(*x*) and *f*_*A*_(*x*), respectively, project each point in *X* to each real number in the interval [0,1], namely, *τ*_*A*_(*p*) : *P*⟶[0,1] and *λ*_*A*_(*p*) : *P*⟶[0,1], where 0⩽*τ*_*A*_(*p*)+*λ*_*A*_(*p*)⩽1, and *δ*_*A*_(*p*)=1 − *τ*_*A*_(*p*) − *λ*(*p*) is called the vague degree of element *p* relative to a vague set *A*, which represents the uncertainty of element *p* relative to vague set *A*. Let *A* be a vague set, and we have *A*=∫_*P*_[*τ*_*A*_(*p*), 1 − *λ*_*A*_(*p*)]/*p* *p* ∈ *P*, when *P* is a continuous space. When *P* is a discrete space, then *A*=∑_*i*=1_^*n*^[*t*_*A*_(*x*_*i*_), 1 − *f*_*A*_(*x*_*i*_)]/*x*_*i*_ *x*_*i*_ ∈ *X*.

**Definition 2 (see [28]).**

Let *p* ∈ *P*, and then, we can define the vague value of set *A* at point *p* as [*τ*_*A*_(*p*), 1 − *λ*_*A*_(*p*)].(*τ*_*A*_(*p*), *λ*_*A*_(*p*), *δ*_*A*_(*p*)), *A*=[*τ*_*A*_(*p*), 1−*λ*_*A*_(*p*)]=[0.4, 1 − 0.4].

**Definition 3 .**

Set vague values *a*=[*τ*_*a*_, 1 − *λ*_*a*_] and *b*=[*τ*_*b*_, 1−*λ*_*b*_], where *τ*_*a*_, *λ*_*a*_, *τ*_*b*_, *λ*_*b*_ ∈ [0,1] and *τ*_*x*_+*λ*_*y*_⩽1, and then, we can define the vague values as follows:

(1) $$\begin{array}{c} a=b\Leftrightarrow \tau_{a}=\tau_{b},\lambda_{a}=\lambda_{b},\\ a⩽b\Leftrightarrow \tau_{a}⩽\tau_{b},\lambda_{a}>\lambda_{b},\\ \bar{a}=\left[\lambda_{a},1-\tau_{a}\right]. \end{array}$$

The description that the attribute value is the vague value in the MADM problem based on the vague set is given as follows:

Let *O*={*O*_1_, *O*_2_,…, *O*_*n*_} be a decision set, and let *R*={*R*_1_, *R*_2_,…, *R*_*m*_} be the corresponding set of attributes; then, the decision matrix of the scheme *O*_*i*_ under the attribute set *R* is expressed by the vague value as

(2) $$\begin{array}{c} A=\left[\begin{array}{cccc} \left(R_{1},\left[\tau_{11},1-\lambda_{11}\right]\right) & \left(R_{2},\left[\tau_{12},1-\lambda_{12}\right]\right) & \cdots & \left(R_{m},\left[\tau_{1m},1-\lambda_{1m}\right]\right)\\ \left(R_{1},\left[\tau_{21},1-\lambda_{21}\right]\right) & \left(R_{2},\left[\tau_{22},1-\lambda_{22}\right]\right) & \cdots & \left(R_{m},\left[\tau_{2m},1-\lambda_{2m}\right)\right.\\ \vdots & \vdots & \cdots & \vdots \\ \left(R_{1},\left[\tau_{n1},1-\lambda_{n1}\right]\right) & \left(R_{2},\left[\tau_{n2},1-\lambda_{n2}\right]\right) & \cdots & \left(R_{m},\left[\tau_{mm},1-\lambda_{mm}\right]\right) \end{array}\right], \end{array}$$

where *τ*_*ij*_ shows the in what extent that scheme, *O*_*i*_ supports the attribute, and *R*_*j*_ represents the disagreement degree that *O*_*i*_ is relative to *R*_*j*_.

#### 2.1.2. Definition and New Calculation Method of Entropy of Vague Set

The entropy is a measurement that represents the fuzziness and information content of the vague set. The definition and a new calculation method are given below.

**Definition 4 .**

We call function F： *V*_*S*_(*p*)⟶[0,1] the entropy of vague set *V*_*S*_(*p*) when it satisfies the following conditions:

1. *F*(*S*)=0⇔*S* is a nonfuzzy set.
2. *F*(*S*)=1⇔ for any *x* ∈ *S*, *τ*_*S*_(*p*)=*λ*_*S*_(*p*)=0.
3. For any vague set *S* defined on P， *F*(*S*)=*F*(*S*^*C*^).
4. For vague set *A* and *B*, when |*τ*_*A*_(*p*) − *λ*_*A*_(*p*)|=|*τ*_*B*_(*p*) − *λ*_*B*_(*p*)| and *δ*_*A*_(*p*) > *δ*_*B*_(*p*)，we have *F*(*A*) > *F*(*B*).
5. For vague sets *A* and *B*, when *δ*_*A*_(*p*)=*δ*_*B*_(*p*)，if ∀*p*_*i*_ ∈ *P*，then |*τ*_*A*_(*p*_*i*_) − *λ*_*A*_(*p*_*i*_)|⩽|*π*_*B*_(*p*_*i*_) − *λ*_*B*_(*p*_*i*_)|, so we have *F*(*A*)⩾*F*(*B*).

According to the five conditions above, a new calculation method of the entropy of a vague set is proposed in the following content.

Let *P*={*p*_1_, *p*_2_, *p*_3_,…, *p*_*n*_} and *A*=∑_*i*=1_^*n*^[*τ*_*A*_(*p*_*i*_), 1 − *λ*_*A*_(*p*_*i*_)]/*p*_*i*_, and then, the entropy of vague set A can be expressed as *F*(*A*)=1/*n*∑_*i*=1_^*n*^1 − |*τ*_*A*_(*p*_*i*_) − *λ*_*A*_(*p*_*i*_)|*e*^|*τ*_*A*_(*p*_*i*_) − *λ*_*A*_(*p*_*i*_)|−1^+*δ*_*A*_(*p*_*i*_)/2.

It can be easily verified that the entropy defined satisfies the abovementioned conditions. Moreover, we can see that *F*(*A*) implied both the fuzziness |*τ*_*A*_(*p*_*i*_) − *λ*_*A*_(*p*_*i*_)|*e*^|*τ*_*A*_(*p*_*i*_) − *λ*_*A*_(*p*_*i*_)|−1^ and the uncertainty *δ*_*A*_(*p*_*i*_).

#### 2.1.3. VEWF-TOPSIS Method and Its Workflow

When the attribute value is vague, the entropy of the vague set is used to measure the influence of a certain evaluation index on the MDAM scheme. The larger the entropy value of each scheme under the attribute *j*, the objective weight of each attribute means that each scheme knows less information on this attribute, or it is more difficult to make a reasonable choice. Normalization is used as the objective weight of the attribute, and the latter case is taken here, and the objective weight of each attribute can be expressed as *θ*_*i*_=1 − *F*_*j*_/*n* − ∑_*j*=1_^*n*^*F*_*j*_ *i*=1,2,…, *m*.

The main steps of the VEWF-TOPSIS method are demonstrated as follows:

(S1) Constructing the vague set decision matrix **A**.
(S2) Calculating the entropy *F* and the objective weight of each attribute *θ*_*i*_, respectively.
(S3) Calculating the negative and positive ideal schemes. When the criterion is a benefit criterion, the positive and negative ideal schemes [29] can be expressed, respectively, as **A**^+^=([*τ*_*i*1_^+^, 1 − *λ*_*i*1_^+^], [*τ*_*i*2_+, 1 − *λ*_*i*2_^+^], [*τ*_*i*3_^+^, 1 − *λ*_*i*3_^+^],…, [*τ*_*im*_^+^, 1 − *λ*_*im*_^+^]) and **A**^−^=([*τ*_*i*1_^−^, 1 − *λ*_*i*1_^−^], [*τ*_*i*2_^−^, 1 − *λ*_*i*2_^−^], [*τ*_*i*3_^−^, 1 − *λ*_*i*3_^−^], ⋯, [*τ*_*im*_^−^, 1 − *λ*_*im*_^−^]).
  When the criterion is a cost criterion, the positive and negative ideal schemes are, respectively, as **A**^+^=([*τ*_*i*1_^−^, 1 − *λ*_*i*1_^−^], [*τ*_*i*2_^−^, 1 − *λ*_*i*2_^−^], [*τ*_*i*3_^−^, 1 − *λ*_*i*3_^−^],…, [*τ*_*im*_^−^, 1 − *λ*_*im*_^−^]). and **A**^−^=([*τ*_*i*1_^+^, 1 − *λ*_*i*1_^+^], [*τ*_*i*2_+, 1 − *λ*_*i*2_^+^], [*τ*_*i*3_^+^, 1 − *λ*_*i*3_^+^],…, [*τ*_*im*_^+^, 1 − *λ*_*im*_^+^])) , where $\left|\tau_{ij}^{+}-\lambda_{ij}^{+}\right|=\underset{i=1,2,\text{…},n}{\max}\left|\tau_{ij}-\lambda_{ij}\right|$, $\left|\tau_{ij}^{+}-\lambda_{ij}^{+}\right|=\underset{i=1,2,\text{…},n}{\min}\left|\tau_{ij}-\lambda_{ij}\right|$. When |*τ*_*ij*_ − *λ*_*ij*_|=|*τ*_*kj*_ − *λ*_*kj*_| and in the situation of benefit criterion, if *τ*_*ij*_ > *τ*_*kj*_，then [*τ*_*ij*_, 1 − *λ*_*ij*_] is better than [*τ*_*kj*_, 1 − *λ*_*kj*_], while in the case of the cost criterion, the opposite is true.
(S4) Calculating the standard weighted Hamming distance between each positive ideal scheme and the negative ideal scheme, respectively, as *d*_*i*_^+^=1/2*m*∑_*j*=1_^*m*^*θ*_*j*_(|*τ*_*ij*_ − *τ*_*j*_^+^|+|*λ*_*ij*_ − *λ*_*j*_^+^|+|*δ*_*ij*_ − *δ*_*j*_^+^|) and *d*_*i*_^−^=1/2*m*∑_*j*=1_^*m*^*θ*_*j*_(|*τ*_*ij*_ − *τ*_*j*_^−^|+|*λ*_*ij*_ − *λ*_*j*_^−^|+|*δ*_*ij*_ − *δ*_*j*_^−^|).
(S5) Calculating the relative closeness of each scheme as *C*_*i*_=*d*_*i*_^−^/*d*_*i*_^+^+*d*_*i*_^−^.

### 2.2. EILIF-TOPSIS method [30]

#### 2.2.1. Definition of iIFS

**Definition 5 .**

Let Θ be a nonempty set, and Φ={<*σ*, *ξ*_Φ_(*σ*), *ς*_Φ_(*σ*)>*|σ* ∈ Φ} is an iIFS defined on Θ, in which *ξ*_Φ_(*σ*) : Φ⟶int(0,1) and *ς*_Φ_(*σ*) : *X*⟶int(0,1) are membership and nonmembership of *σ* ∈ Φ and *σ* ∉ Φ. The functions *ξ*_*A*_(*x*) and *ς*_*A*_(*x*), essentially, need to be subjected to the constraint condition that 0 ≤ sup(*ξ*_Φ_(*σ*))+sup(*ς*_Φ_(*σ*)) ≤ 1. In addition, when we need to show the hesitation degree, then *θ*_Φ_(*σ*)=1 − *ξ*_Φ_(*σ*) − *ς*_Φ_(*σ*) is defined.

Furthermore, we set *ξ*_Φ_(*σ*)=[*ξ*_Φ_^−^(*σ*), *ξ*_Φ_^+^(*σ*)] and *ς*_Φ_(*σ*)=[*ς*_Φ_^−^(*σ*), *ς*_Φ_^+^(*σ*)], and then, the iIFS has the form of Φ={〈*σ*, [*ξ*_Φ_^−^(*σ*), *ξ*_Φ_^+^(*σ*)], [*ς*_Φ_^−^(*σ*), *ς*_Φ_^+^(*σ*)]〉*|σ* ∈ Φ}. Correspondingly, we define *θ*_Φ_(*σ*)=[*θ*_Φ_^−^(*σ*), *θ*_Φ_^+^(*σ*)]=[1 − *ξ*_Φ_^+^(*σ*) − *ς*_Φ_^+^(*σ*), 1 − *ξ*_Φ_^−^(*σ*) − *ς*_Φ_^−^(*σ*)] as the hesitation degree for *σ* ∈ Φ.

**Definition 6 (see [31]).**

We call *α*=([*ξ*_*α*_^−^, *ξ*_*α*_^+^], [*ς*_*α*_^−^, *ς*_*α*_^+^]) an interval-valued intuitionistic number, in which [*μ*_*α*_^−^, *μ*_*α*_^+^] ⊂ [0,1], [*v*_*α*_^−^, *v*_*α*_^+^] ⊂ [0,1], 0 ≤ *μ*_*α*_^+^+*v*_*α*_^+^ ≤ 1. Obviously, the largest and smallest interval-valued intuitionistic numbers are *α*^+^=([1,1], [0,0]) and *α*^−^=([0,0], [1,1]), respectively.

**Definition 7 .**

If *α*=([*ξ*_*α*_^−^, *ξ*_*α*_^+^], [*ς*_*α*_^−^, *ς*_*α*_^+^]) is an interval-valued intuitionistic number, then we define the scoring function for *α* as given by *s*(*α*)=1/2(*ξ*_*α*_^−^ − *ς*_*α*_^−^+*ξ*_*α*_^+^ − *ς*_*α*_^+^), in which *s*(*α*) ∈ [−1,1].

#### 2.2.2. Similarity of iIFSs

**Definition 8 (see [32]).**

Suppose that *H*: *H*(*X*) × *H*(*X*)⟶[0,1] is a real-valued function, and then *H* can be seen as a similarity function defined on an iIFS A when the conditions listed in [32] are satisfied.

**Definition 9 (see [33]).**

Suppose that *X*={*x*_1_, *x*_2_,…, *x*_*n*_} is a finite universe. For two iIFSs *A* and *B*, reference [33] uses the definition of entropy to construct the formula of similarity between *A* and *B* and simplifies it. The similarity *S*(*A*, *B*) of *A* and *B* has the calculation formula of *S*(*A*, *B*)=1/*n*∑_*j*=1_^*n*^2 − min{*ξ*_*j*_^−^, *ς*_*j*_^−^} − min{*ξ*_*j*_^+^, *ς*_*j*_^+^}/2+max{*ξ*_*j*_^−^, *ς*_*j*_^−^}+max{*ξ*_*j*_^+^, *ς*_*j*_^+^}, in which *ξ*_*j*_^−^=|*ξ*_*A*_^−^(*x*_*j*_) − *ξ*_*B*_^−^(*x*_*j*_)|, *ς*_*j*_^−^=|*ς*_*A*_^−^(*x*_*j*_) − *ς*_*B*_^−^(*x*_*j*_)|, *ξ*_*j*_^+^=|*ξ*_*A*_^+^(*x*_*j*_) − *ξ*_*B*_^+^(*x*_*j*_)|, *ς*_*j*_^+^=|*ς*_*A*_^+^(*x*_*j*_) − *ς*_*B*_^+^(*x*_*j*_)|.

The validity of the similarity formula given above has been thoroughly verified in the existing literature [30]. Meanwhile, the weighted similarity of two iIFSs *A* and *B* is given as

(3) $$\begin{array}{c} S\left(A,B\right)=\sum_{j=1}^{n}w_{j}2-\min\left\{\xi_{j}^{-},\varsigma_{j}^{-}\right\}-\min\left\{\xi_{j}^{+},\varsigma_{j}^{+}\right\}/2+\max\left\{\xi_{j}^{-},\varsigma_{j}^{-}\right\}+\max\left\{\xi_{j}^{+},\varsigma_{j}^{+}\right\}, \end{array}$$

in which *w*_*j*_ represents the element of the weight matrix, and

(4) $$\begin{array}{c} \xi_{j}^{-}=\left|\xi_{A}^{-}\left(x_{j}\right)-\xi_{B}^{-}\left(x_{j}\right)\right|,\;\varsigma_{j}^{-}=\left|\varsigma_{A}^{-}\left(x_{j}\right)-\varsigma_{B}^{-}\left(x_{j}\right)\right|,\\ \xi_{j}^{+}=\left|\xi_{A}^{+}\left(x_{j}\right)-\xi_{B}^{+}\left(x_{j}\right)\right|,\;\varsigma_{j}^{+}=\left|\varsigma_{A}^{+}\left(x_{j}\right)-\varsigma_{B}^{+}\left(x_{j}\right)\right|. \end{array}$$

#### 2.2.3. Principles and Workflow of EILIF-TOPSIS Method

It is clear that obtaining the entropy of attribute value is of most importance in the entropy-weighted method, and in the interval intuitionistic fuzzy environment, the entropy of attribute value has also been given a new meaning. Since the score function of an iIFN reflects the degree of hesitation, that is, the degree of fuzziness and the average information entropy of each attribute can be calculated from the score function, then the weight value of related attributes can be calculated. Obviously, in MADM problems, when the weight information is unknown, this algorithm is much simpler and easier to be put into practice than the traditional algorithms that require solving a single objective optimization model.

When Shannon created information theory [34], he defined the entropy of a discrete source as follows:

(5) $$\begin{array}{c} F:\left[\begin{array}{cccc} x_{1} & x_{2} & \cdots & x_{n}\\ p_{1} & p_{2} & \cdots & p_{n} \end{array}\right], \end{array}$$

where the priori probability of random variable *F* is *p*_*i*_, 0 ≤ *p*_*i*_ ≤ 1, ∑*p*_*i*_=1 , *i*=1,2,…, *n*, and the uncertainty of the source is described by the prior probability distribution *P*={*p*_1_, *p*_2_,…, *p*_*n*_}*s*; thus, the average uncertainty of the information source can be given as *H*_*s*_(*P*)=−*k*∑_*i*=1_^*n*^*p*_*i*_log  *p*_*i*_ , in which the constant *k* depends on the selected unit, usually *k* = 1, and the base of the logarithmic function in the above formula is usually 2, 10, or *e*. In this section, *e* is chosen as the base of the logarithmic function. With this idea, Shannon's information entropy is extended to the mean information entropy of interval-valued intuitionistic fuzzy numbers using the scoring function value.

For the interval-valued intuitionistic fuzzy decision matrix, *F*=[*α*_*ij*_]_*n*×*m*_, where *α*_*ij*_=(*u*_*ij*_, *v*_*ij*_). For each scheme *A*_*i*_(*i*=1,2,…, *n*), we solve the score function value of *Ai* about the feature information of attribute *Xj* and then normalize it, so that $\tilde{s}\left(\alpha_{ij}\right)=s\left(\alpha_{ij}\right)/\sum_{i=1}^{n}s\left(\alpha_{ij}\right)$.

Then, use above formula to find the average information entropy of each attribute *Xj*

(6) $$\begin{array}{c} H_{s}\left(X_{j}\right)=-\frac{1}{\ln\;n}\sum_{i=1}^{n}\tilde{s}\left(\alpha_{ij}\right)\ln\tilde{s}\left(\alpha_{ij}\right). \end{array}$$

We say that when $\tilde{s}\left(\alpha_{ij}\right)=0$，the relation $\tilde{s}\left(\alpha_{ij}\right)\ln\tilde{s}\left(\alpha_{ij}\right)=0$ is satisfied. The following formula is used to find the weight:

(7) $$\begin{array}{c} \omega_{j}=\frac{1-H_{s}\left(X_{j}\right)}{\sum_{k=1}^{m}\left(1-H_{s}\left(X_{k}\right)\right)}\;,j=1,2,\text{…},m. \end{array}$$

When obtaining the weights, the final solution ranking can be calculated by using the iIF-TOPSIS method.

The basic principle of the TOPSIS method can be explained as follows. Suppose that there are *n* alternative decision-making schemes that form a scheme set *A*={*A*_1_, *A*_2_,…, *A*_*n*_}, and each scheme has *m* attributes *X*={*X*_1_, *X*_2_,…, *X*_*n*_}. Let *u*_*ij*_=[*u*_*ij*_^−^, *u*_*ij*_^+^] ⊂ [0,1] and *v*_*ij*_=[*v*_*ij*_^−^, *v*_*ij*_^+^] ⊂ [0,1] indicate the degrees that the scheme *A*_*i*_ ∈ *A* satisfies and does not satisfy the attribute *X*_*j*_ ∈ *X*, where 0 ≤ *u*_*ij*_^+^+*v*_*ij*_^+^ ≤ 1. That is, the evaluation of the scheme *Ai* with respect to the attribute *Xj* is available in the iIFS *F*_*ij*_=(*u*_*ij*_, *v*_*ij*_): *A*_*i*_=(*F*_*i*1_, *F*_*i*2_, *F*_*i*3_,…, *F*_*im*_)={[*u*_*i*1_, *v*_*i*1_], [*u*_*i*2_, *v*_*i*2_], [*u*_*i*3_, *v*_*i*3_]…[*u*_*im*_, *v*_*im*_]}.

According to the basic idea of the TOPSIS method, the calculation steps of the iIF-TOPSIS method are given as follows:

(S1) Determine the positive ideal scheme *A*^+^ and the negative ideal scheme *A*^−^:
  (8) $$\begin{array}{c} A^{+}=\left\{\langle \left[u_{1+}^{-},u_{1+}^{+}\right],\left[v_{1+}^{-},v_{1+}^{+}\right]\rangle ,\text{…},\langle \left[u_{n+}^{-},u_{n+}^{+}\right],\left[v_{n+}^{-},v_{n+}^{+}\right]\rangle \right\},\\ A^{-}=\left\{\langle \left[u_{1-}^{-},u_{1-}^{+}\right],\left[v_{1-}^{-},v_{1-}^{+}\right]\rangle ,\cdots ,\langle \left[u_{n-}^{-},u_{n-}^{+}\right],\left[v_{n-}^{-},v_{n-}^{+}\right]\rangle \right\}, \end{array}$$
where for ∀*j*=1,2,…, *n*
  (9) $$\begin{array}{c} \langle \left[u_{j+}^{-},u_{j+}^{+}\right],\left[v_{j+}^{-},v_{j+}^{+}\right]\rangle =\langle \left[\max_{i}u_{ij}^{-},\max_{i}u_{ij}^{+}\right],\left[\min_{i}v_{ij}^{-},\min_{i}v_{ij}^{+}\right]\rangle ,\\ \langle \left[u_{j-}^{-},u_{j-}^{+}\right],\left[v_{j-}^{-},v_{j-}^{+}\right]\rangle =\langle \left[\min_{i}u_{ij}^{-},\min_{i}u_{ij}^{+}\right],\left[\max_{i}v_{ij}^{-},\max_{i}v_{ij}^{+}\right]\rangle . \end{array}$$
(S2) Normalize the score function value of each attribute to obtain the final score function matrix $S={\left(\tilde{s}\left(\alpha_{ij}\right)\right)}_{n\times m}$.
(S3) Calculate the average information content of the output of each attribute: when $\tilde{s}\left(\alpha_{ij}\right)=0$ is specified, $\tilde{s}\left(\alpha_{ij}\right)\ln\tilde{s}\left(\alpha_{ij}\right)=0$.
(S4) Calculate the weight coefficient *w*_*j*_,  *j*=1,2,…, *n* of each attribute *X*_*j*_.
(S5) Calculate the similarity functions *S*(*A*_*i*_, *A*^+^) and *S*(*A*_*i*_, *A*^−^).
(S6) Calculate the relative similarity: *S*(*A*_*i*_)=*S*(*A*_*i*_, *A*^+^)/*S*(*A*_*i*_, *A*^+^)+*S*(*A*_*i*_, *A*^−^) , *i*=1,2,…, *n*.
(S7) Determine the ordering of the scenarios in decreasing trend.

### 2.3. IEAI-IF-TOPSIS Method

#### 2.3.1. Attribute Importance Based on Information Entropy in Intuitive Fuzzy Information System

In practical problems, the descriptions of some objects are often reflected as IFNs. For example, when voting for a candidate, it is divided into yes, no, and abstentions, and the voting result can be expressed as an intuitive fuzzy number.

Suppose that *A*_1_, *A*_2_,…, *A*_*m*_ are *m* alternatives, and *C*_1_, *C*_2_,…*C*_*n*_ are *n* corresponding attributes, in which (*μ*_*ij*_, *v*_*ij*_)(*i*=1,2,…, *m*; *j*=1,2,…, *n*) are intuitionistic fuzzy numbers, representing the evaluation value of the program *Ai* under the attribute *Cj*. All evaluation values constitute an intuitionistic fuzzy information system. In MADM problems, the inherent information of each scheme can be used to obtain the information of the attribute entropy [35].

Let *p*_*ij*_=*μ*_*ij*_/∑_*i*=1_^*m*^*μ*_*ij*_, *q*_*ij*_=*v*_*ij*_/∑_*i*=1_^*m*^*v*_*ij*_, in which *p*_*ij*_ is the membership coefficient; *q*_*ij*_ is the nonmembership coefficient. We call *E*_*j*_=−1/2  ln  *m*∑_*i*=1_^*m*^(*p*_*ij*_ln  *p*_*ij*_+*q*_*ij*_ln  *q*_*ij*_) the information entropy of the *j*-th attribute *Cj*, representing the total contribution of the *m*-th alternative *Am* to the *j*-th attribute *Cj*. It is easy to see that 0 ≤ *Ej* ≤ 1. In particular, when *p*_*ij*_=*q*_*ij*_=1/*m*, *E*_*j*_=1.

It can be seen that as the membership and nonmembership coefficients tend to be consistent, *Ej* tends to 1; especially when the membership contribution and nonmembership contribution are congruent, it can be different. Consider the role of this attribute in decision-making; that is, the weight of the attribute should be 0 at this time. We call *d*_*j*_=1 − *E*_*j*_ the attribute importance of the *j*-th attribute *Cj*. Furthermore, the attribute weight of the *j*-th attribute *Cj* can be defined as *ω*_*j*_=*d*_*j*_/∑_*j*=1_^*n*^*d*_*j*_. It should be easy to see that ∑_*j*=1_^*n*^*ω*_*j*_=1. The feature of this method is that, in the process of MADM, the information provided by the information system can be used to calculate the weight of the attribute without introducing the subjective judgment of the decision maker.

#### 2.3.2. TOPSIS Method Based on Intuitionistic Fuzzy Information Entropy Importance

Suppose that a MADM problem has *m* alternatives *O*_1_, *O*_2_,…, *O*_*m*_ and *n* attributes *Q*_1_, *Q*_2_,…, *Q*_*n*_. The evaluation value of each option under each attribute constitutes a decision matrix.

The decision-making process of the TOPSIS method based on the importance of intuitionistic fuzzy information entropy is as follows:

(S1) Compute the normative decision matrix, whose normative values are
  (10) $$\begin{array}{c} n_{ij}=\frac{x_{ij}}{\sqrt{\sum_{i=1}^{m}x_{ij}^{2}}},\;j=1,2,\text{…},n. \end{array}$$
(S2) Calculate the weighted normative decision matrix.
(S3) Determine the positive and negative ideal solutions.
(S4) Calculate the separation of each scenario from the positive and negative ideal solutions.
(S5) Calculate the relative closeness *r*_*i*_^*∗*^ of an alternative to a positive ideal solution.
(S6) Sort the alternatives according to *r*_*i*_^*∗*^ from large to small, determine the pros and cons of the alternatives, and finally decide which one should be chosen.

It can be seen from the classic TOPSIS method that, in the MADM problems, the weight of the attribute is of great importance. In the intuitionistic fuzzy information system, the method of determining the attribute weight according to the information entropy is to first determine the attribute weight, and then establish a method based on the intuitionistic fuzzy information system:

(S1) Establish evaluation criteria (attributes) and propose alternative solutions.
(S2) Evaluate each solution according to the criteria, and its evaluation value is expressed as an intuitionistic fuzzy number.
(S3) Construct an intuitionistic fuzzy decision matrix, and the elements in the intuitionistic fuzzy decision matrix, in which *α*_*ij*_=(*μ*_*ij*_, *v*_*ij*_) are intuitionistic fuzzy numbers.
(S4) Calculate the weighted intuitionistic fuzzy decision matrix, its weighted intuitionistic fuzzy value ${\tilde{\alpha }}_{ij}=\omega_{j}\cdot \alpha_{ij}=\left({\tilde{\mu }}_{ij},{\tilde{v}}_{ij}\right)$, and *ω*_*j*_ is the weight of the attribute *Cj* determined by the information entropy method.
(S6) Determine intuitive fuzzy positive and negative ideal solutions:
  (11) $$\begin{array}{c} {\tilde{A}}^{+}=\left\{\alpha_{1}^{+},\alpha_{2}^{+},\text{…},\alpha_{n}^{+}\right\}=\left\{\left[\left(\max_{i}{\tilde{\mu }}_{ij},\min_{i}{\tilde{v}}_{ij}\right)|j\in I\right],\left[\left(\min_{i}{\tilde{\mu }}_{ij},\max_{i}{\tilde{v}}_{ij}\right)|j\in J\right]\right\},\\ {\tilde{A}}^{-}=\left\{\alpha_{1}^{-},\alpha_{2}^{-},\text{…},\alpha_{n}^{-}\right\}=\left\{\left[\left(\min_{i}{\tilde{\mu }}_{ij},\max_{i}{\tilde{v}}_{ij}\right)|j\in I\right],\left[\left(\max_{i}{\tilde{\mu }}_{ij},\min_{i}{\tilde{v}}_{ij}\right)|j\in J\right]\right\}, \end{array}$$
where *I* represents the set of beneficial attribute, and *J* stands for the set of cost attribute.
(S6) Calculate the degree of separation between the alternatives and the positive and negative ideal solutions using the distance formula between intuitionistic fuzzy numbers: ${\tilde{d}}_{i}^{+}=\sum_{j=1}^{n}d\left({\tilde{\alpha }}_{ij},\alpha_{j}^{+}\right),{\tilde{d}}_{i}^{-}=\sum_{j=1}^{n}d\left({\tilde{\alpha }}_{ij},\alpha_{j}^{-}\right)$.
(S7) Calculate the relative closeness of an alternative to a positive ideal solution: ${\tilde{r}}_{i}={\tilde{d}}_{i}^{-}/{\tilde{d}}_{i}^{+}+{\tilde{d}}_{i}^{-}\left(i=1,2,\text{…},m\right)$.
(S8) Sort the alternatives according to ${\tilde{r}}_{i}$ from largest to smallest, and make a final decision.

### 2.4. Comprehensive Approach for MADM Problem

In the process of practical application, model uncertainty and subjectivity of expert experience are common. In the above three kinds of methods, there are certain fuzziness and uncertainty in both the model selection and the weight interval given by experts. Therefore, using only one of these methods will inevitably lead to the inaccuracy or objectivity of the analysis results. When a single method is used for investment decision-making of large-scale projects, the unreasonable of a small link will greatly reduce the investment effect.

In order to improve the shortcomings of the aforementioned methods in terms of the objectivity of indicators, the objectivity of expert scoring, and the rationality of method selection, based on the idea of weighted synthesis and the relative proximity of the ideal solution of the decision-making optimization scheme as the parameter, the results obtained by the aforementioned three types of methods were analyzed. Comprehensive quantification is carried out to obtain a comprehensive ideal solution ranking, and the final ideal solution is optimized. This paper adopts the following formula:

(12) $$\begin{array}{c} {\tilde{R}}_{i}=\sum_{j=1}^{p}\omega_{ij}{\tilde{r}}_{j}, \end{array}$$

where ${\tilde{R}}_{i}$、 *ω*_*ij*_ and ${\tilde{r}}_{j}$ are the comprehensive closeness, weight, and relative closeness mentioned above.

In real-world problems, the abovementioned weight coefficient is the key to conduct the proposed comprehensive approach. Here, we need to point out that when determining the values of *ω*_*ij*_, methods such as AHP and ELECTRE should be used to integrate multiple suggestions from numerous experts in relative investment decision-making projects. As for ${\tilde{r}}_{j}$, the relative closeness obtained by the abovementioned method will be sufficient to be utilized. Only in this way can we solve the problems of large-scale project investment properly; that is, the manpower and money cost in the project can be minimized.

## 3. Case Study and Discussions

We take the problem of address selection for a supermarket that invested by a certain Company A for case study. In this case, if the supermarket wants to make a profit, it can only operate in a low-cost way. For example, it employs fewer personnel than the department store, and the service is not as good as the department store. According to environmental trends, influencing events, and the needs of the industry, the future distribution industry will be in an international and diversified market, market segmentation, industry homogeneity, and operational obstacles will increase, and international standards will increase. The impact of laws and international brands will increase, so that the industry will face more and greater challenges.

**Figure 3:**
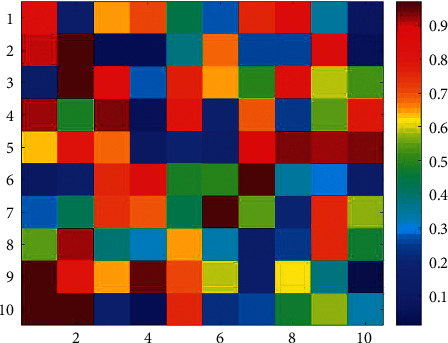
Mid-value map of the decision-making matrix.

Company A was used to focus on logistics. Recently, the company intends to invest in a supermarket chain project with an investment amount of 1 billion yuan. In the early stage of project investment, the company initially formulated a qualitative project investment plan on the premise of soliciting opinions from various parties, but it still needs to conduct quantitative analysis on the location selection of the supermarket chain. In this numerical case, we suppose that the company needs to make a decision on the best address of one of the supermarkets to be invested in. The address to be selected is *P*1∼*P*10, and the factors affecting the address selection include 10 types of factors such as residential density, traffic convenience, and community safety, represented by *R*1∼*R*9. The method proposed in this paper is used to quantify the selection of supermarket addresses. The decision-making matrix that the experts made is illustrated in Table 1 and Figure 3.

**Figure 4:**
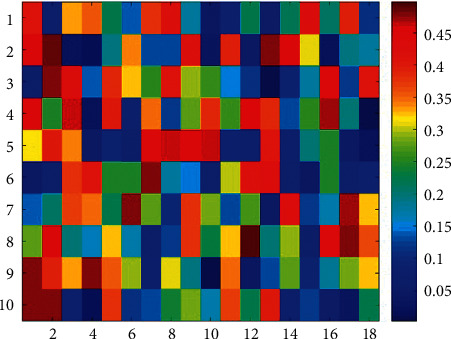
Mid-value map of the membership and nonmembership contributions.

**Figure 5:**
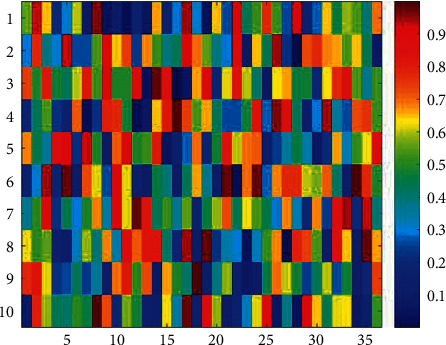
Mid-value map of the interval-valued intuitionistic fuzzy decision-making matrix.

The membership and nonmembership contributions in the method of EILIF-TOPSIS are given in Table 2 and Figure 4, and the interval-valued intuitionistic fuzzy decision-making matrix in IEAI-IF-TOPSIS is given in Figure 5.

**Figure 6:**
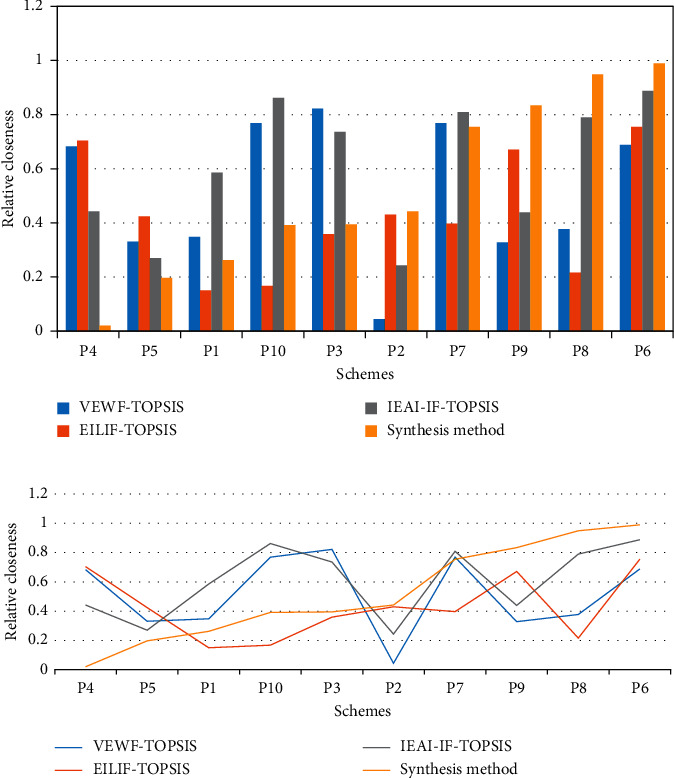
Relative closeness calculation results obtained by the four methods. (a) Histogram of relative closeness calculation results obtained by the four methods. (b) Line chart of relative closeness calculation results obtained by the four methods.

**Figure 7:**
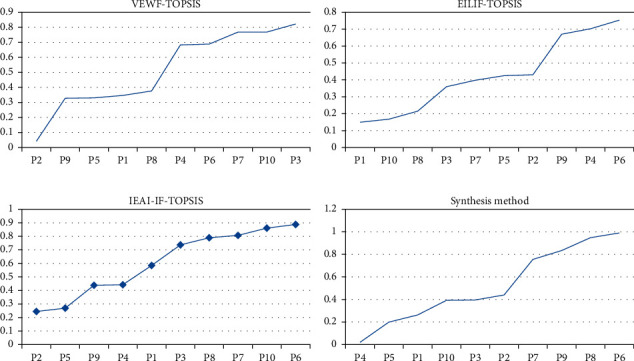
Ordered relative closeness calculation results obtained by the four methods. (a) VEWF-TOPSIS results. (b) EILIF-TOPSIS results. (c) IEAI-IF-TOPSIS results. (d) Synthesis method results.

Based on the calculation framework of the VEWF-TOPSIS, EILIF-TOPSIS, and IEAI-IF-TOPSIS methods and the proposed synthesis method, the orders of relative closeness are given in Table. 3, Figures 6 and 7. From the calculation results of the synthesis method, we can see that the supermarket investment decision scheme P6 is the best among all the alternatives. Further, it can be seen from Figure 6 that the proposed synthesis method has a certain degree of “compromise,” which can reduce the model uncertainty and the subjectivity of expert experience of the above three methods. The optimal scheme obtained by the proposed method is also consistent with the schemes obtained by the other three methods, which proves the rationality of the proposed method.

## 4. Conclusions

To effectively handle the MADM problems related to most of the large-scale project investment items, in the present work, we take advantages of three most popular MADM frameworks, namely, VEWF-TOPSIS, EILIF-TOPSIS, and IEAI-IF-TOPSIS methods, and reconcile these methods in a systematic way. The contribution of this paper is that, by synthesizing the three abovementioned methods, the subjectivity of single source expert experience can be effectively avoided on the basis of further reducing the uncertainty of the model. In the process of actual large-scale project investment decision-making, using the method proposed in this paper can effectively reduce the investment risk and improve the investment income. The research results and conclusions mainly include the following:

1. According to the characteristics and development status of large-scale project investment processes, the risk factors, including technical risk, social risk, management risk, economic risk, natural environment risk, and institutional risk, should be considered thoroughly and systematically.
2. From the viewpoint of objectiveness, the IEAI-IF-TOPSIS method tends to be a better MADM framework for large-scale problems due to the quantitative of the fuzzy weights. However, these three methods are essentially subjective techniques, so that, in real-world MAMD problems, we should use all the possible manners systematically to reach the goal of successful project investment.
3. The method proposed in this paper combines the advantages of VEWF-TOPSIS, EILIF-TOPSIS, and IEAI-IF-TOPSIS and has compromise and conservatism. The optimal scheme obtained is in good agreement with the other three types of methods, and the advantages and disadvantages of the other schemes are the synthesis of the results obtained by the other three types of methods. Therefore, in real-world investment decision-making problems, the proposed method can effectively reduce the investment risk and make the investment return higher.
4. In real-world large-scale project investment problems, we should have the correct guiding ideology; it is necessary to clarify why to invest, the link that most needs investment, its own conditions and resources, and the market environment. Meanwhile, we should have an overall concept. It is necessary to consider the combination of immediate interests and long-term interests and avoid the unfavorable situation that may affect the overall and long-term development of the enterprise caused by “short-term and short-sightedness.”

MADM problems not only exist in large-scale project investment, but also commonly happen in the fields of product design, human resource management, etc. The future work of the authors may focus on applying the proposed method to the MADM problems in the abovementioned fields, and more advanced method will be studied based on the presented framework.

## Figures and Tables

**Table 1:** Decision-making matrix of the supermarket address selection problem.

Schemes	*R*1	*R*2	*R*3	*R*4	*R*5	*R*6	*R*7	*R*8	*R*9

*P*1	[0.3, 0.5]	[0.3, 0.7]	[0.2, 0.6]	[0.1, 0.5]	[0.1, 0.5]	[0.2, 0.6]	[0.2, 0.7]	[0.5, 0.6]	[0.2, 0.5]
*P*2	[0.2, 0.5]	[0.2, 0.5]	[0.1, 0.5]	[0.3, 0.6]	[0.3, 0.6]	[0.1, 0.6]	[0.1, 0.6]	[0.4, 0.7]	[0.4, 0.5]
*P*3	[0.4, 0.5]	[0.2, 0.5]	[0.3, 0.7]	[0.3, 0.5]	[0.4, 0.8]	[0.4, 0.5]	[0.4, 0.5]	[0.3, 0.8]	[0.5, 0.6]
*P*4	[0.3, 0.7]	[0.4, 0.5]	[0.3, 0.6]	[0.3, 0.4]	[0.3, 0.9]	[0.3, 0.7]	[0.3, 0.8]	[0.2, 0.5]	[0.1, 0.3]
*P*5	[0.4, 0.8]	[0.1, 0.5]	[0.5, 0.8]	[0.4, 0.6]	[0.2, 0.7]	[0.3, 0.8]	[0.4, 0.9]	[0.5, 0.7]	[0.2, 0.6]
*P*6	[0.6, 0.9]	[0.3, 0.6]	[0.4, 0.9]	[0.3, 0.7]	[0.1, 0.7]	[0.2, 0.6]	[0.1, 0.4]	[0.1, 0.4]	[0.2, 0.5]
*P*7	[0.2, 0.6]	[0.7, 0.9]	[0.2, 0.6]	[0.4, 0.8]	[0.4, 0.7]	[0.4, 0.7]	[0.2, 0.3]	[0.4, 0.6]	[0.4, 0.7]
*P*8	[0.4, 0.5]	[0.6, 0.8]	[0.3, 0.9]	[0.3, 0.7]	[0.3, 0.6]	[0.1, 0.5]	[0.4, 0.6]	[0.3, 0.5]	[0.5, 0.6]
*P*9	[0.2, 0.4]	[0.2, 0.6]	[0.1, 0.7]	[0.1, 0.6]	[0.1, 0.5]	[0.3, 0.8]	[0.2, 0.5]	[0.5, 0.8]	[0.2, 0.5]
*P*10	[0.1, 0.7]	[0.1, 0.5]	[0.2, 0.5]	[0.2, 0.5]	[0.2, 0.4]	[0.4, 0.9]	[0.1, 0.4]	[0.6, 0.7]	[0.1, 0.7]

**Table 2:** Membership and nonmembership contributions in the method of EILIF-TOPSIS.

Schemes	*R*1	*R*2	*R*3	*R*4	*R*5	*R*6	*R*7	*R*8	*R*9

*P*1	(0.4, 0.1)	(0.3, 0.4)	(0.2, 0.1)	(0.4, 0.4)	(0.2, 0.3)	(0.1, 0.2)	(0.1, 0.2)	(0.4, 0.2)	(0.4, 0.1)
*P*2	(0.5, 0.5)	(0.1, 0.3)	(0.2, 0.3)	(0.1, 0.1)	(0.4, 0.2)	(0.4, 0.2)	(0.5, 0.3)	(0.3, 0.1)	(0.2, 0.2)
*P*3	(0.1, 0.5)	(0.4, 0.1)	(0.4, 0.3)	(0.3, 0.4)	(0.3, 0.3)	(0.2, 0.1)	(0.3, 0.1)	(0.2, 0.5)	(0.1, 0.4)
*P*4	0.5, 0.2)	(0.5, 0.0)	(0.4, 0.1)	(0.3, 0.1)	(0.3, 0.4)	(0.3, 0.5)	(0.4, 0.1)	(0.3, 0.5)	(0.2, 0.2)
*P*5	(0.3, 0.4)	(0.3, 0.1)	(0.1, 0.1)	(0.4, 0.5)	(0.5, 0.5)	(0.1, 0.1)	(0.4, 0.1)	(0.2, 0.2)	(0.3, 0.5)
*P*6	(0.3, 0.1)	(0.4, 0.4)	(0.2, 0.2)	(0.5, 0.2)	(0.1, 0.1)	(0.3, 0.4)	(0.4, 0.1)	(0.1, 0.2)	(0.1, 0.1)
*P*7	(0.1, 0.2)	(0.4, 0.3)	(0.2, 0.5)	(0.3, 0.1)	(0.4, 0.3)	(0.1, 0.3)	(0.1, 0.4)	(0.1, 0.2)	(0.5, 0.3)
*P*8	(0.3, 0.5)	(0.2, 0.2)	(0.3, 0.2)	(0.1, 0.1)	(0.4, 0.2)	(0.3, 0.5)	(0.2, 0.3)	(0.1, 0.5)	(0.5, 0.4)
*P*9	(0.5, 0.4)	(0.3, 0.5)	(0.4, 0.3)	(0.1, 0.3)	(0.2, 0.4)	(0.3, 0.1)	(0.1, 0.3)	(0.1, 0.2)	(0.3, 0.3)
*P*10	(0.5, 0.5)	(0.1, 0.2)	(0.4, 0.1)	(0.1, 0.2)	(0.3, 0.2)	(0.4, 0.2)	(0.4, 0.1)	(0.1, 0.1)	(0.3, 0.2)

**Table 3:** Calculation results of the relative closeness obtained from the four methods.

Methods	*P*1	*P*2	*P*3	*P*4	*P*5	*P*6	*P*7	*P*8	*P*9	*P*10

VEWF-TOPSIS	0.348	0.044	0.822	0.683	0.331	0.688	0.769	0.377	0.328	0.769
EILIF-TOPSIS	0.150	0.430	0.359	0.704	0.424	0.755	0.397	0.216	0.671	0.167
IEAI-IF-TOPSIS	0.586	0.243	0.736	0.442	0.270	0.888	0.809	0.790	0.439	0.862
Synthesis method	0.262	0.442	0.395	0.020	0.197	0.990	0.755	0.949	0.834	0.391

## Data Availability

The data used to support the findings of this study are available from the corresponding author upon request.
